# Mini-Review of Studies Testing the Cardiorespiratory Hypothesis With Near-Infrared Spectroscopy (NIRS): Overview and Perspectives

**DOI:** 10.3389/fnins.2021.699948

**Published:** 2021-08-12

**Authors:** Nounagnon Frutueux Agbangla, Pauline Maillot, Damien Vitiello

**Affiliations:** ^1^Laboratory I3SP (URP 3625), Institute of Sport and Health Sciences of Paris – Université de Paris/UFR STAPS, Paris, France; ^2^Laboratory URePSSS - SHERPAS (ULR 7369), Univ. Artois, Univ. Littoral Côte d’Opale, Univ. Lille/UFR STAPS, Liévin, France

**Keywords:** near-infrared spectroscopy, cardiorespiratory fitness, executive functions, hemodynamic activity, brain

## Abstract

The cardiorespiratory hypothesis (CH) is one of the hypotheses used by researchers to explain the relationship between cardiorespiratory fitness and cognitive performance during executive functions. Despite the indubitable beneficial effect of training on brain blood flow and function that may explain the link between physical fitness and cognition and the recognition of the near-infrared spectroscopy (NIRS) as a reliable tool for measuring brain oxygenation, few studies investigated the CH with NIRS. It is still not well understood whether an increase in brain flow by training is translated into an increase in cerebral oxygenation. Thus, the objective of this mini-review was to summarize main results of studies that investigated the CH using the NIRS and to propose future research directions.

## Introduction

A growing body of evidence shows that physical exercise has a beneficial effect on cognition (Gomes-Osman et al., 2018; Loprinzi et al., 2018; Herold et al., 2019; Netz, 2019; Sanders et al., 2019). Nevertheless, the effects of physical exercise on cognition are heterogeneous, as they are more pronounced on executive functions (Andrés and Van der Linden, 2000; Fjell et al., 2014). Several hypotheses, namely the cardiorespiratory hypothesis (CH), neurogenesis increase hypothesis, synaptic plasticity increase hypothesis, catecholamines increase hypothesis, and cognitive enrichment hypothesis are used to explain these cognitive effects of physical exercise (Audiffren et al., 2011; Maillot and Perrot, 2012). Beyond these hypotheses, many researchers are using the CH to explain their findings regarding the effect of exercise on cognition. The CH suggests that the improvement in cognitive performance observed in subjects following an aerobic program could be due to an increase in cerebral metabolic activity (Dustman et al., 1984). The increase in metabolic activity induces increased oxygen and glucose transportation to the brain. In other words, the CH stipulates those subjects who have a high cardiorespiratory fitness oxygenate the brain areas involved in cognitive tasks more efficiently, which allows them to better perform tasks (e.g., executive tasks). Thus, to validate this hypothesis, it is necessary to measure cerebral oxygenation. One of the tools able to measure this oxygenation in a non-invasive way is the near-infrared spectroscopy (NIRS). The biological mechanisms (angiogenesis, vascular plasticity, and better vascular health) underlying the CH were presented in a recent review (see Agbangla et al., 2019b). Some studies have tested this hypothesis by examining the relationship between cognitive performance, changes in brain blood flow as measured by functional magnetic resonance imaging (fMRI) and cardiorespiratory fitness (VO_2_max) following an aerobic program (Colcombe et al., 2004; Pereira et al., 2007). Their results showed that participants not only increase their cerebral blood flow in different arteries (i.e., middle frontal gyrus, superior parietal cortex, and dentate gyrus), but also their VO_2_max. In addition, it was demonstrated that the enhancement in cerebral blood flow was related to a significant or substantial improvement of cognitive performance (i.e., selective attention task, verbal learning, and memory) and cardiorespiratory fitness. Thus, studies using fMRI provide undeniable evidence of the CH but do not allow us to know whether the increase in brain flow translates into an increase in oxygen, which is essential for brain metabolism (Attwell et al., 2010).

To answer this question, some authors used NIRS as a tool to examine cerebral hemodynamic activity in humans (Chance et al., 1993; Hoshi and Tamura, 1993; Kato et al., 1993; Villringer et al., 1993), which reflects the neurovascular coupling mechanism that induces both an increase in local cerebral blood volume and blood flow (Villringer and Chance, 1997). Indeed, the NIRS allows the investigation of hemodynamic changes associated with brain activity evaluated by changes in oxyhemoglobin (O_2_Hb), deoxyhemoglobin (HHb), and total hemoglobin (HbT) concentration (Agbangla et al., 2017). Hemodynamic parameters provided by NIRS (i.e., O_2_Hb, HHb, HbT) while can be considered as more specific for cerebral oxygenation compared to the fMRI-BOLD signal, the raw NIRS-signals cannot be taken as measures for hemodynamic parameters such as flow, volume, oxygen extraction function. Hence it is essential to rely on mathematical models to determine these parameters like the ones reported by Kocsis et al. (2006) and Mukli et al. (2018). Several reviews have outlined the principles, strengths, limitations, and good practices of the NIRS (Ekkekakis, 2009; Scholkmann et al., 2014; Yücel et al., 2021). Despite the physiological and methodological validation of NIRS, a meticulous search of various databases shows that few studies have investigated the CH using the NIRS even though it has been recognized as a reliable tool for measuring brain oxygenation. In this context, the objective of this mini-review was, to summarize the key results of the studies that investigated the CH using the NIRS.

## Literature Search

**FIGURE 1:**
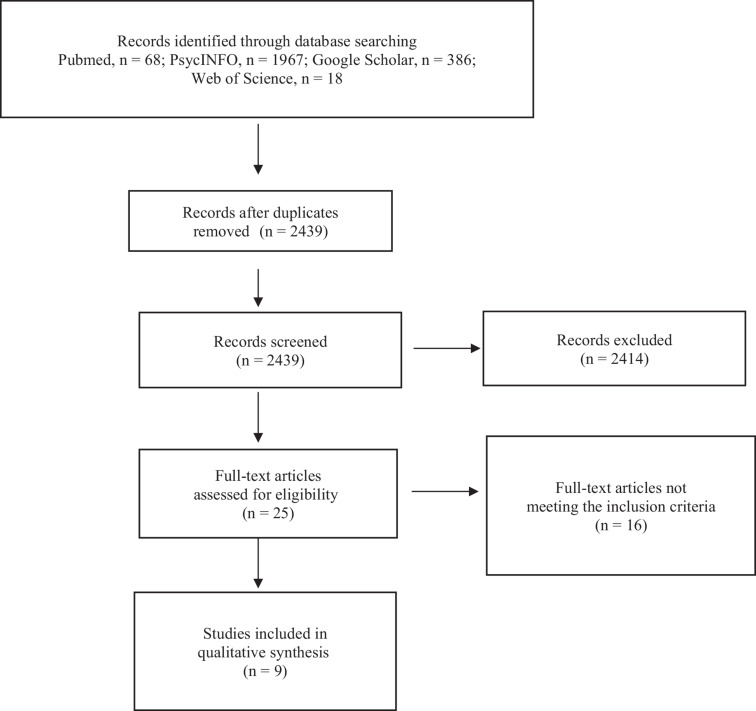
Chart flow of the selection process.

To perform this mini-review, the Google Scholar, Pubmed, PsycINFO, and Web of Science databases were searched (last search on March 2021) using the terms “cardiovascular fitness” OR “cardiorespiratory fitness” OR “aerobic fitness” AND “fNIRS” OR “NIRS” AND “executive functions.” The search strategy retrieved 386 articles on Google Scholar, 68 articles on Pubmed, 1967 articles on PsycINFO, and 18 articles on Web of Science. Following this search, duplicate references were removed. After this identification step, we proceeded to the screening step, which involved sifting through the titles and abstracts to check their relevance. Studies were selected if they were conducted on populations of any age and had cardiorespiratory fitness and/or executive functions and/or hemodynamic activity as parameters of interest. Twenty-five articles were selected, and their full texts were reviewed by authors for inclusion in the mini-systematic review. Only studies that tested the CH were included. Following the expertise of the selected articles, nine articles were considered in the writing of the mini-review (Figure 1).

## Participants and Variables of Interest

### Participants

Except for the subject in the case study by Cabral et al. (2017) who had been addicted to alcohol and cigarettes for 33 years, subjects of the different studies were either young adults between 17 and 25 years or older adults over 60 years. In terms of cardiorespiratory fitness, studies compared subjects who were highly fit or active with their counterparts who had a low fit or were inactive.

### Executive Functions

Executive functions (EFs) are cognitive processes that regulate thought and action in unusual situations (Friedman et al., 2006). More precisely, there are high-level cognitive processes that, through their influence on lower-level processes, enable individuals to regulate their thoughts and actions during goal-directed behavior (Friedman and Miyake, 2017; p. 1). They can be subdivided into core EFs, namely inhibition, updating of working memory, shifting, and higher-order EFs such as reasoning, problem-solving, planning, and dual-task management (Miyake et al., 2000; Diamond, 2013). To measure executive functions, studies that investigated the CH used various executive tasks such as the Stroop task (Dupuy et al., 2015; Cabral et al., 2017; Kujach et al., 2018; Ludyga et al., 2019; Goenarjo et al., 2020a), random number generation (Albinet et al., 2014), n-back (Agbangla et al., 2019a), the dual-task (Goenarjo et al., 2020b), and the Trail Making Test (TMT) (Mekari et al., 2019). These executive tasks allowed the researchers to measure, respectively, the interference control, behavioral inhibition, updating of working memory, shifting, and dual-task management. In brief, the studies cited above used more tasks involving core EFs than tasks requiring higher-order EFs. Finally, only two studies considered the complexity of the executive task (Albinet et al., 2014; Agbangla et al., 2019a). Indeed, the study by Albinet et al. (2014) used the random number generation with two paces that consisted, firstly, of giving a digit every second and a half and, secondly, a digit every second. In a study by Agbangla and colleagues, three levels of complexity of the n-back were proposed to the subjects (i.e., 1, 2, 3-back) (Agbangla et al., 2019a).

### Cardiorespiratory Fitness

Cardiorespiratory fitness is the ability to perform a dynamic activity of moderate to high intensity involving large-muscle groups over a prolonged period (American College of Sports Medicine (ACSM), 2006). It is highly dependent on the integrity of the cardiovascular, respiratory, and musculoskeletal systems (Hayes et al., 2013). Cardiorespiratory fitness is measured by several methods. The reference method is the maximal effort test in which the participant is asked to perform a physical effort with an incremental load (Hayes et al., 2013). The maximal oxygen consumption is then determined by gas analysis. In default of this gold standard test, sub-maximal tests and questionnaires can be used to estimate cardiorespiratory fitness. Most studies that have investigated the CH have used the maximal effort test (Albinet et al., 2014; Dupuy et al., 2015; Cabral et al., 2017; Kujach et al., 2018; Mekari et al., 2019; Goenarjo et al.,2020a,b). However, other studies have used a sub-maximal test (Ludyga et al., 2019) or a sub-maximal test and questionnaire (Agbangla et al., 2019a). These tests allowed the researchers to measure various cardiorespiratory parameters such as VO_2_max, peak oxygen consumption (VO_2_peak), peak power output, and relative power output to muscle mass.

### Brain Hemodynamic Activity

All studies measure hemodynamic activity in the prefrontal cortex (PFC) regions (i.e., left and right dorsolateral, left and right ventrolateral, frontopolar, and anterior prefrontal cortices) using continuous wave NIRS. However, unlike frequency and time domain NIRS, continuous wave NIRS does not allow for absolute measurements of hemodynamic activity (Scholkmann et al., 2014). According to these authors, continuous wave NIRS does not fully determine the scattering and absorption coefficients of near-infrared light. To determine hemodynamic activity, the authors mainly used the subtractive method, which involves subtracting the concentration of the hemodynamic index (HHb and/or O_2_Hb, and/or HbT) during the baseline or compatible condition from the concentration of the hemodynamic index during the experimental or incompatible condition (Dupuy et al., 2015; Kujach et al., 2018; Agbangla et al., 2019a; Ludyga et al., 2019; Mekari et al., 2019; Goenarjo et al.,2020a,b). Apart from this subtractive method, another method, consisting of applying a linear regression on the entire signal window to obtain the slope coefficient of the regression line (slope method), was used in a study by Albinet et al. (2014).

## Links Between Executive Functions, Cardiorespiratory Fitness, and Cerebral Hemodynamic Activity

Cross-sectional studies showed that young and old adults with high (46–56 mL^–1^kg^–1^min^–1^ for young and 26–30 mL^–1^kg^–1^min^–1^ for old adults) cardiorespiratory fitness have better executive performance (i.e., updating of working memory, inhibition, shifting, and dual-task ability) compared to their counterparts with low (36–38 mL^–1^kg^–1^ min^–1^ for young and 17–21 mL^–1^kg^–1^ min^–1^ for old adults) cardiorespiratory fitness (Albinet et al., 2014; Dupuy et al., 2015; Agbangla et al., 2019a; Ludyga et al., 2019; Mekari et al., 2019; Goenarjo et al.,2020a,b). These results have also been confirmed by intervention and case studies, showing that participants who followed an aerobic program or an intermittent high-intensity session improved their executive performance (Cabral et al., 2017; Kujach et al., 2018) and their cardiorespiratory fitness (Cabral et al., 2017). Another important finding is that some of the studies included in the review highlighted a link between executive performance and cardiorespiratory fitness or cerebral hemodynamic. Indeed, some of the cross-sectional studies reported that only participants (young and older adults) with high cardiorespiratory fitness significantly increase their cerebral hemodynamic activity during the executive task (Dupuy et al., 2015; Goenarjo et al., 2020a). In addition, other cross-sectional studies reported that participants with high cardiorespiratory fitness who increased their cerebral hemodynamic activity did the best executive performances (Agbangla et al., 2019a; Mekari et al., 2019). These results suggest a link between cardiorespiratory fitness, cerebral hemodynamic activity and executive performance. Finally, other authors have observed a correlation between cardiorespiratory fitness and executive performance in older adults (Albinet et al., 2014) and young adults (Ludyga et al., 2019). However, this correlation was mediated by hemodynamic activity only in older adults. These results concur with the results of studies that have tested the CH using fMRI (Colcombe et al., 2004; Pereira et al., 2007). These results suggest that the effect of cardiorespiratory fitness on executive functions is mediated by an increase in hemodynamic activity depending on the age of the participant. The mediating effect of hemodynamic activity on the link between physical exercise and executive performance could be explained by the fact that physical exercise increases cerebral blood flow (Zimmerman et al., 2014). Indeed, the increase in cerebral blood flow ensures the supply of the necessary energetic substrates and oxygen to neuronal networks involved in the investigated executive function. However, the increase in cerebral blood flow would be underpinned by several biological mechanisms. On the one hand, we have angiogenesis, enhanced vascular plasticity, and improved vascular health (Agbangla et al., 2019b). Regarding angiogenesis, regular exercise induces the release of neurotrophic factors, namely vascular endothelial growth factor and insulin growth factor 1, which are required for angiogenesis (Lopez-Lopez et al., 2004; Audiffren et al., 2011). As for vascular plasticity, it is underpinned by a better bioavailability of nitric oxide which enhances vasodilatation (Tanaka et al., 2000). Finally, the improvement in vascular health is explained by the beneficial effects of exercise on arterial stiffness and vascular dysfunction (Albinet et al., 2008). On the other hand, the increase in blood flow could be explained by the modulation of functional brain networks during the performance of the cognitive task (Racz et al., 2017; Kaposzta et al., 2021). This modulation would result in a redistribution of local support systems (i.e., blood flow, oxygenation, and metabolism). In this context, we can hypothesized that participants with good cardiorespiratory fitness would be the ones with better modulation of functional brain networks most likely due to enhanced neuro-vascular coupling.

## Futures Perspectives of Research

The summary of studies presented in this mini-review showed that the subjects solicited in the exploration of the CH were generally healthy young (17–25 years) or older (over 60 years). Consequently, consideration of other age groups between 25 and 59 years but also pathological populations is essential for extending the CH to other populations. For example, it would be interesting to explore this hypothesis in patients with cerebrovascular pathologies. A recent meta-analysis showed that a program combining aerobic and resistance exercise (i.e., performed at moderate intensity, three times a week for 20 weeks) had a greater effect on cardiorespiratory fitness, muscle strength, and walking ability in stroke patients (Lee and Stone, 2020) than aerobic or resistance training alone. However, this effect of exercise on cardiorespiratory fitness is not related to executive functions, which are dysfunctional in 75% of stroke patients (Povroznik et al., 2018). Hence, future studies may explore the links between executive functions, cardiorespiratory fitness and cerebral oxygenation in stroke patients and even in other populations suffering from other chronic pathologies using a randomized controlled protocol. From this perspective, several interventions can be suggested to participants according to their functional abilities. For example, fit participants may participate in a training program combining endurance and resistance (Lee and Stone, 2020) or in a training program combining physical and cognitive exercises (Ji et al., 2019). Moreover, the immersive virtual reality could be proposed to participants with low functional capacity preventing them from doing traditional physical exercise (Burin et al., 2020). At last, these future studies in patients with stroke or other chronic conditions should also consider the motivational profile of the patients to adjust the physical activity program. Indeed, patients with high self-efficacy, expectancy and self-determination are those who are more likely to be involved in a physical activity program and to maintain physical activity (Sweet et al., 2011). However, when dealing with unmotivated patients, the use of a motivational intervention would be appropriate to induce engagement in the physical activity program (Schertz et al., 2019).

Various executive tasks have been used to measure executive performance. Among these tasks, there is the Stroop task, the random number generation test, the n-back test, the Trail Making Test, and the dual-task test. However, the limitation of these tasks is their inability to directly measure an executive function without involving other cognitive processes (Miyake et al., 2000). Indeed, confirmatory analyses performed on the main components of executive functioning (i.e., inhibition, updating of working memory, and shifting) showed that inhibition had a common factor with the rest of the executive functions (Miyake and Friedman, 2012). Thus, inhibition would be found in several executive tasks that would require the suppression of responses, distractors, and memory representations (Zacks and Hasher, 1994). To address this problem of impurity in neuropsychological tasks that measure executive functions, some authors have used multiple tasks to assess an executive function (Albinet et al., 2012; Boucard et al., 2012). This multi-task method for assessing executive function appears to be adequate in our opinion and should be used in future studies exploring the cardiorespiratory fitness hypothesis to obtain a composite score of executive functioning.

Finally, despite the diversity of the executive functions assessed in the different studies considered in this mini-review, all studies measured hemodynamic activity only in the PFC. Although it is undoubtedly recognized that the PFC is invariably involved in executive functioning (Collette et al., 2006). However, other brain areas are activated when performing an executive task. For example, the random number generation task is associated with significant activation of the left dorsolateral PFC, anterior cingulate cortex, superior parietal cortex, inferior frontal cortex, and right and left cerebellar hemispheres (Jahanshahi et al., 2000). In the Stroop task, the areas activated include the dorsolateral PFC, the supplementary motor area, the left premotor cortex, the superior temporal gyrus, the left putamen, and the anterior cingulate cortex (Pardo et al., 1990; Banich, 2009). Regarding N-back, brain imaging studies have shown activation of the right dorsolateral PFC, the anterior cingulate cortex, the posterior parietal cortex, and the inferior frontal gyrus (Collette et al., 2006). As for the Trail Making Test, it has been shown that the left dorsolateral PFC, supplementary motor area, cingulate sulcus, intraparietal sulcus are the areas that are activated (Moll et al., 2002). Finally, the dual-task comprised a cognitive-motor task soliciting not only the PFC but also the sensorimotor area, the supplementary motor area, and the occipital cortex (Van Impe et al., 2011). When we identified the brain areas that were activated during the executive tasks used in the CH, we found that there were three brain areas that were common to the tasks (i.e., PFC, parietal cortex, cingulate cortex). Therefore, it is essential to extend the measurement of the hemodynamic activity to the parietal and cingulate cortices to explore the connectivity of these areas with the PFC in the context of the CH. It would be judicious to explore the interactions between the various physiological systems (i.e., central nervous, respiratory, cardiovascular systems) involved in the CH and executive performances using the integrative approach (Bashan et al., 2012; Mukli et al., 2021) in future studies. It will be a question, for example, of exploring both the nature and the strength of the interactions that take place between these different physiological systems and the effects of these interactions on executive performances.

## Conclusion

This mini-review highlights that the majority of studies exploring the cardiorespiratory fitness hypothesis enrolled healthy young and old participants. These studies are CH mostly cross-sectional studies. Therefore, it is suggested that randomized controlled studies conducted in other age groups (e.g., 8–16 or 25–59 years old) or in participant with different pathological concerns (e.g., stroke; cognitive disorders), using a multi-task method may offer a valuable way to measure executive functions and answer to the CH.

## Author Contributions

NA and DV contributed to conception and design of the review, selected and read the studies included in the review, and organized the database based on the review of literature. NA wrote the first draft of the manuscript. NA, PM, and DV wrote sections of the manuscript. All authors contributed to manuscript revision, read, and approved the submitted version.

## Conflict of Interest

The authors declare that the research was conducted in the absence of any commercial or financial relationships that could be construed as a potential conflict of interest.

## Publisher’s Note

All claims expressed in this article are solely those of the authors and do not necessarily represent those of their affiliated organizations, or those of the publisher, the editors and the reviewers. Any product that may be evaluated in this article, or claim that may be made by its manufacturer, is not guaranteed or endorsed by the publisher.

## References

[B1] Agbangla N. F. Audiffren M. Albinet C. T. (2017). Use of near-infrared spectroscopy in the investigation of brain activation during cognitive aging: a systematic review of an emerging area of research. *Ageing Res. Rev.* 38 52–66. 10.1016/j.arr.2017.07.003 28755870

[B2] Agbangla N. F. Fraser S. A. Albinet C. T. (2019b). An Overview of the Cardiorespiratory Hypothesis and Its Potential Contribution to the Care of Neurodegenerative Disease in Africa. *Medicina* 55:601. 10.3390/medicina55090601 31533346 PMC6780802

[B3] Agbangla N. F. Audiffren M. Pylouster J. Albinet C. T. (2019a). Working Memory, Cognitive Load and Cardiorespiratory Fitness: testing the CRUNCH Model with Near-Infrared Spectroscopy. *Brain Sci.* 9:38. 10.3390/brainsci9020038 30744137 PMC6406418

[B4] Albinet C. Fezzani K. Thon B. V. (2008). Vieillissement, activité physique et cognition. *Mov. Sport Sci.* 63 9–36. 10.3917/sm.063.0009 18052372

[B5] Albinet C. T. Boucard G. Bouquet C. A. Audiffren M. (2012). Processing speed and executive functions in cognitive aging: how to disentangle their mutual relationship? *Brain Cogn.* 79 1–11. 10.1016/j.bandc.2012.02.001 22387275

[B6] Albinet C. T. Mandrick K. Bernard P. L. Perrey S. Blain H. (2014). Improved cerebral oxygenation response and executive performance as a function of cardiorespiratory fitness in older women: a fNIRS study. *Front. Aging Neurosci.* 6:272. 10.3389/fnagi.2014.00272 25339900 PMC4189417

[B7] American College of Sports Medicine (ACSM). (2006). *ACSM’s Guidelines for Exercise Testing and Prescription*, 7th Edn. Philadelphia: Lippincott Williams & Wilks’ins.

[B8] Andrés P. Van der Linden M. (2000). Age-related differences in supervisory attentional system functions. *J. Gerontol. B Psychol. Sci. Soc. Sci.* 55 373–380. 10.1093/geronb/55.6.p373 11078107

[B9] Attwell D. Buchan A. M. Charpak S. Lauritzen M. Macvicar B. A. Newman E. A. (2010). Glial and neuronal control of brain blood flow. *Nature* 468 232–243. 10.1038/nature09613 21068832 PMC3206737

[B10] Audiffren M. André N. Albinet C. T. (2011). Positive effects of chronic physical exercise on cognitive functions in aging people: assessment and prospects. *Revue de Neuropsychol.* 3 207–225.

[B11] Banich M. T. (2009). Executive function. The search for an integrated account. *Curr. Dir. in Psychol. Sci.* 18 89–94. 10.1111/j.1467-8721.2009.01615.x

[B12] Bashan A. Bartsch R. P. Kantelhardt J. W. Havlin S. Ivanov P. (2012). Network physiology reveals relations between network topology and physiological function. *Nat. Commun.* 3:702. 10.1038/ncomms1705 22426223 PMC3518900

[B13] Boucard G. K. Albinet C. T. Bugaiska A. Bouquet C. A. Clarys D. Audiffren M. (2012). Impact of physical activity on executive functions in aging: a selective effect on inhibition among old adults. *J. Sport Exerc. Psychol.* 34 808–827. 10.1123/jsep.34.6.808 23204360

[B14] Burin D. Liu Y. Yamaya N. Kawashima R. (2020). Virtual training leads to physical, cognitive and neural benefits in healthy adults. *NeuroImage* 222:117297. 10.1016/j.neuroimage.2020.117297 32828927

[B15] Cabral D. A. da Costa K. G. Okano A. H. Elsangedy H. M. Rachetti V. P. Fontes E. B. (2017). Improving cerebral oxygenation, cognition and autonomic nervous system control of a chronic alcohol abuser through a three-month running program. *Addict. Behav. Rep.* 6 83–89. 10.1016/j.abrep.2017.08.004 29450240 PMC5800586

[B16] Chance B. Zhuang Z. UnAh C. Alter C. Lipton L. (1993). Cognition-activated low-frequency modulation of light absorption in human brain. *Proc. Natl. Acad. Sci. U. S. A.* 90 3770–3774. 10.1073/pnas.90.8.3770 8475128 PMC46383

[B17] Colcombe S. J. Kramer A. F. Erickson K. I. Scalf P. McAuley E. Cohen N. J. (2004). Cardiovascular fitness, cortical plasticity, and aging. *Proc. Natl. Acad. Sci. U. S. A.* 101 3316–3321. 10.1073/pnas.0400266101 14978288 PMC373255

[B18] Collette F. Hogge M. Salmon E. Van der Linden M. (2006). Exploration of the neural substrates of executive functioning by functional neuroimaging. *Neuroscience* 139 209–221. 10.1016/j.neuroscience.2005.05.035 16324796

[B19] Diamond A. (2013). Executive functions. *Annu. Rev. Psychol.* 64 135–168. 10.1146/annurev-psych-113011-143750 23020641 PMC4084861

[B20] Dupuy O. Gauthier C. J. Fraser S. A. Desjardins-Crèpeau L. Desjardins M. Mekary S. (2015). Higher levels of cardiovascular fitness are associated with better executive function and prefrontal oxygenation in younger and older women. *Front. Hum. Neurosci.* 9:66. 10.3389/fnhum.2015.00066 25741267 PMC4332308

[B21] Dustman R. E. Ruhling R. O. Russell E. M. Shearer D. E. Bonekat H. W. Shigeoka J. W. (1984). Aerobic exercise training and improved neuropsychological function of older individuals. *Neurobiol. Aging* 5 35–42. 10.1016/0197-4580(84)90083-6 6738784

[B22] Ekkekakis P. (2009). Illuminating the black box: investigating prefrontal cortical hemodynamics during exercise with near-infrared spectroscopy. *J. Sport Exerc. Psychol.* 31 505–553. 10.1123/jsep.31.4.505 19842545

[B23] Fjell A. M. McEvoy L. Holland D. Dale A. M. Walhovd K. B. Alzheimer’s Disease (2014). What is normal in normal aging? Effects of aging, amyloid and Alzheimer’s disease on the cerebral cortex and the hippocampus. *Prog. Neurobiol.* 117 20–40. 10.1016/j.pneurobio.2014.02.004 24548606 PMC4343307

[B24] Friedman N. P. Miyake A. (2017). Unity and diversity of executive functions: individual differences as a window on cognitive structure. *Cortex* 86 186–204. 10.1016/j.cortex.2016.04.023 27251123 PMC5104682

[B25] Friedman N. P. Miyake A. Corley R. P. Young S. E. Defries J. C. Hewitt J. K. (2006). Not all executive functions are related to intelligence. *Psychol. Sci.* 17 172–179. 10.1111/j.1467-9280.2006.01681.x 16466426

[B26] Goenarjo R. Dupuy O. Fraser S. Perrochon A. Berryman N. Bosquet L. (2020b). Cardiorespiratory fitness, blood pressure, and cerebral oxygenation during a dual-task in healthy young males. *Behav. Brain Res.* 380:112422. 10.1016/j.bbr.2019.112422 31837344

[B27] Goenarjo R. Bosquet L. Berryman N. Metier V. Perrochon A. Fraser S. A. (2020a). Cerebral Oxygenation Reserve: the Relationship Between Physical Activity Level and the Cognitive Load During a Stroop Task in Healthy Young Males. *Int. J. Environ. Res. Public Health* 17:1406. 10.3390/ijerph17041406 32098221 PMC7068614

[B28] Gomes-Osman J. Cabral D. F. Morris T. P. McInerney K. Cahalin L. P. Rundek T. (2018). Exercise for cognitive brain health in aging: a systematic review for an evaluation of dose. *Neurology. Clin. Pract.* 8 257–265. 10.1212/CPJ.0000000000000460 30105166 PMC6075983

[B29] Hayes S. M. Hayes J. P. Cadden M. Verfaellie M. (2013). A review of cardiorespiratory fitness-related neuroplasticity in the aging brain. *Front. Aging Neurosci.* 5:31. 10.3389/fnagi.2013.00031 23874299 PMC3709413

[B30] Herold F. Müller P. Gronwald T. Müller N. G. (2019). Dose-Response Matters! - A Perspective on the Exercise Prescription in Exercise-Cognition Research. *Front. Psychol.* 10:2338. 10.3389/fpsyg.2019.02338 31736815 PMC6839278

[B31] Hoshi Y. Tamura M. (1993). Detection of dynamic changes in cerebral oxygenation coupled to neuronal function during mental work in man. *Neurosci. Lett.* 150 5–8. 10.1016/0304-3940(93)90094-2 8469403

[B32] Jahanshahi M. Dirnberger G. Fuller R. Frith C. D. (2000). The role of the dorsolateral prefrontal cortex in random number generation: a study with positron emission tomography. *Neuroimage* 12 713–725. 10.1006/nimg.2000.0647 11112403

[B33] Ji Z. Feng T. Mei L. Li A. Zhang C. (2019). Influence of acute combined physical and cognitive exercise on cognitive function: an NIRS study. *PeerJ.* 7:e7418. 10.7717/peerj.7418 31396453 PMC6681798

[B34] Kaposzta Z. Stylianou O. Mukli P. Eke A. Racz F. S. (2021). Decreased connection density and modularity of functional brain networks during n-back working memory paradigm. *Brain Behav.* 11:e01932. 10.1002/brb3.1932 33185986 PMC7821619

[B35] Kato T. Kamei A. Takashima S. Ozaki T. (1993). Human visual cortical function during photic stimulation monitoring by means of near-infrared spectroscopy. *J. Cereb. Blood Flow Metab.* 13 516–520. 10.1038/jcbfm.1993.66 8478409

[B36] Kocsis L. Herman P. Eke A. (2006). Mathematical model for the estimation of hemodynamic and oxygenation variables by tissue spectroscopy. *J. Theor. Biol.* 241 262–275. 10.1016/j.jtbi.2005.11.033 16413035

[B37] Kujach S. Byun K. Hyodo K. Suwabe K. Fukuie T. Laskowski R. (2018). A transferable high-intensity intermittent exercise improves executive performance in association with dorsolateral prefrontal activation in young adults. *Neuroimage* 169 117–125. 10.1016/j.neuroimage.2017.12.003 29203453

[B38] Lee J. Stone A. J. (2020). Combined Aerobic and Resistance Training for Cardiorespiratory Fitness, Muscle Strength, and Walking Capacity after Stroke: a Systematic Review and Meta-Analysis. *J. Stroke Cerebrovasc. Dis.* 29:104498. 10.1016/j.jstrokecerebrovasdis.2019.104498 31732460

[B39] Lopez-Lopez C. LeRoith D. Torres-Aleman I. (2004). Insulin-like growth factor I is required for vessel remodeling in the adult brain. *Proc. Natl. Acad. Sci. U. S. A.* 101 9833–9838. 10.1073/pnas.0400337101 15210967 PMC470760

[B40] Loprinzi P. D. Edwards M. K. Crush E. Ikuta T. Del Arco A. (2018). Dose-Response Association Between Physical Activity and Cognitive Function in a National Sample of Older Adults. *Am. J. Health Promot.* 32 554–560. 10.1177/0890117116689732 29214828

[B41] Ludyga S. Mücke M. Colledge F. Pühse U. Gerber M. (2019). A Combined EEG-fNIRS Study Investigating Mechanisms Underlying the Association between Aerobic Fitness and Inhibitory Control in Young Adults. *Neuroscience* 419 23–33. 10.1016/j.neuroscience.2019.08.045 31487542

[B42] Maillot P. Perrot A. (2012). La théorie de l’enrichissement cognitif à travers la stimulation physique: activité physique traditionnelle versus exergames. *Neurol. Psychiatrie Gériat.* 12 217–229. 10.1016/j.npg.2012.07.008

[B43] Mekari S. Dupuy O. Martins R. Evans K. Kimmerly D. S. Fraser S. (2019). The effects of cardiorespiratory fitness on executive function and prefrontal oxygenation in older adults. *Geroscience* 41 681–690. 10.1007/s11357-019-00128-5 31728899 PMC6885073

[B44] Miyake A. Friedman N. P. (2012). The nature and organization of individual differences in executive functions: four general conclusions. *Curr. Dir. Psychol. Sci.* 21 8–14. 10.1177/0963721411429458 22773897 PMC3388901

[B45] Miyake A. Friedman N. P. Emerson M. J. Witzki A. H. Howerter A. Wager T. D. (2000). The unity and diversity of executive functions and their contributions to complex “Frontal Lobe” tasks: a latent variable analysis. *Cogn. Psychol.* 41 49–100. 10.1006/cogp.1999.0734 10945922

[B46] Moll J. de Oliveira-Souza R. Moll F. T. Bramati I. E. Andreiuolo P. A. (2002). The cerebral correlates of set-shifting: an fMRI study of the trail making test. *Arq. Neuropsiquiatr.* 60 900–905. 10.1590/s0004-282x2002000600002 12563376

[B47] Mukli P. Nagy Z. Racz F. S. Herman P. Eke A. (2018). Impact of Healthy Aging on Multifractal Hemodynamic Fluctuations in the Human Prefrontal Cortex. *Front. Physiol.* 9:1072. 10.3389/fphys.2018.01072 30147657 PMC6097581

[B48] Mukli P. Nagy Z. Racz F. S. Portoro I. Hartmann A. Stylianou O. (2021). Two-Tiered Response of Cardiorespiratory-Cerebrovascular Network to Orthostatic Challenge. *Front. Physiol.* 12:622569. 10.3389/fphys.2021.622569 33737882 PMC7960776

[B49] Netz Y. (2019). Is There a Preferred Mode of Exercise for Cognition Enhancement in Older Age? *Front. Med.* 6:57. 10.3389/fmed.2019.00057 30984760 PMC6450219

[B50] Pardo J. V. Pardo P. J. Janer K. W. Raichle M. E. (1990). The anterior cingulate cortex mediates processing selection in the Stroop attentional conflict paradigm. *Proc. Natl. Acad. Sci. U S. A.* 87 256–259. 2296583 10.1073/pnas.87.1.256 PMC53241

[B51] Pereira A. C. Huddleston D. E. Brickman A. M. Sosunov A. A. Hen R. McKhann G. M. (2007). An in vivo correlate of exercise-induced neurogenesis in the adult dentate gyrus. *Proc. Natl. Acad. Sci. U. S. A.* 104 5638–5643. 10.1073/pnas.0611721104 17374720 PMC1838482

[B52] Povroznik J. M. Ozga J. E. Vonder Haar C. Engler-Chiurazzi E. B. (2018). Executive (dys)function after stroke: special considerations for behavioral pharmacology. *Behav. Pharmacol.* 29 638–653. 10.1097/FBP.0000000000000432 30215622 PMC6152929

[B53] Racz F. S. Mukli P. Nagy Z. Eke A. (2017). Increased prefrontal cortex connectivity during cognitive challenge assessed by fNIRS imaging. *Biomed. Opt. Express* 8 3842–3855. 10.1364/BOE.8.003842 28856054 PMC5560845

[B54] Sanders L. Hortobágyi T. La Bastide-van G. S. van der Zee E. A. van Heuvelen M. (2019). Dose-response relationship between exercise and cognitive function in older adults with and without cognitive impairment: a systematic review and meta-analysis. *PLoS One* 14:e0210036. 10.1371/journal.pone.0210036 30629631 PMC6328108

[B55] Schertz A. Herbeck Belnap B. Chavanon M. L. Edelmann F. Wachter R. Herrmann-Lingen C. (2019). Motivational interviewing can support physical activity in elderly patients with diastolic heart failure: results from a pilot study. *ESC Heart Fail.* 6 658–666. 10.1002/ehf2.12436 30963721 PMC6676275

[B56] Scholkmann F. Kleiser S. Metz A. J. Zimmermann R. Mata Pavia J. Wolf U. (2014). A review on continuous wave functional near-infrared spectroscopy and imaging instrumentation and methodology. *NeuroImage* 85 6–27. 10.1016/j.neuroimage.2013.05.004 23684868

[B57] Sweet S. N. Tulloch H. Fortier M. S. Pipe A. L. Reid R. D. (2011). Patterns of motivation and ongoing exercise activity in cardiac rehabilitation settings: a 24-month exploration from the TEACH Study. *Ann. Behav. Med.* 42 55–63. 10.1007/s12160-011-9264-2 21374100

[B58] Tanaka H. Dinenno F. A. Monahan K. D. Clevenger C. M. DeSouza C. A. Seals D. R. (2000). Aging, habitual exercise, and dynamic arterial compliance. *Circulation* 102 1270–1275. 10.1161/01.cir.102.11.1270 10982542

[B59] Van Impe A. Coxon J. P. Goble D. J. Wenderoth N. Swinnen S. P. (2011). Age-related changes in brain activation underlying single- and dual-task performance: visuomanual drawing and mental arithmetic. *Neuropsychologia* 49 2400–2409. 10.1016/j.neuropsychologia.2011.04.016 21536055

[B60] Villringer A. Chance B. (1997). Non-invasive optical spectroscopy and imaging of human brain function. *Trends Neurosci.* 20 435–442. 10.1016/S0166-2236(97)01132-6 9347608

[B61] Villringer A. Planck J. Hock C. Schleinkofer L. Dirnagl U. (1993). Near infrared spectroscopy (NIRS): a new tool to study hemodynamic changes during activation of brain function in human adults. *Neurosci. Lett.* 154 101–104. 10.1016/0304-3940(93)90181-j 8361619

[B62] Yücel M. A. Lühmann A. V. Scholkmann F. Gervain J. Dan I. Ayaz H. (2021). Best practices for fNIRS publications. *Neurophotonics* 8:012101. 10.1117/1.NPh.8.1.012101 PMC7793571 33442557

[B63] Zacks R. T. Hasher L. (1994). “Directed ignoring: inhibitory regulation of working memory” in *Inhibitory Processes in Attention, Memory, and Language.* eds Dagenbach D. Carr T. H. (San Diego: Academic Press). 241–264.

[B64] Zimmerman B. Sutton B. P. Low K. A. Fletcher M. A. Tan C. H. Schneider-Garces N. (2014). Cardiorespiratory fitness mediates the effects of aging on cerebral blood flow. *Front. Aging Neurosci.* 6:59. 10.3389/fnagi.2014.00059 24778617 PMC3985032

