# Supplementary material for: Mini-Review of Studies Testing the Cardiorespiratory Hypothesis With Near-Infrared Spectroscopy (NIRS): Overview and Perspectives
Source: Front Neurosci. 2021 Aug 12;15:699948. doi: 10.3389/fnins.2021.699948 (PMC8387658; doi:10.3389/fnins.2021.699948)
Supplement: Supplementary file 1 [file Table_1.docx]

Supplementary Material

| **Table 1 – Studies exploring the cardiorespiratory hypothesis using the NIRS** | | | |
| --- | --- | --- | --- |
| **References** | **Characteristics of study** | **Type of intervention** | **Outcomes** |
| Agbangla et al. (2019) | *Subjects*: 19 younger (19.7±1 years) 37 older adults (68.95±4.74); *Design study*: cross sectional study; *Task*: n-back (0,1,2,3-back); *Executive function*: working memory updating; *Instruments:* OxyMon MkIII; Artinis Medical Systems BV, Zetten, Netherlands); *Cerebral area investigated*: prefrontal regions (left and right dorsolateral and left and right ventrolateral); *Measure of Aerobic fitness*: maximal multistage 20-m shuttle run test (Young) and NASA/JSC physical activity scale (Older adults) - VO_2_max was estimated. | No intervention | - Chronological age had deleterious effects on both cognitive performance and prefrontal cortex activation under a higher cognitive load.  - In older adults, higher levels of cardiorespiratory fitness were related bilateral & prefrontal cortex activation that allowed them to sustain better cognitive performances, especially under the highest cognitive load. |
| Albinet et al. (2014) | *Subjects*: older with high-fit (67.32±4.48) and older with low-fit (68.88±3.87); *Design study*: cross sectional study; *Task*: Random Number Generation (RNG); *Executive function*: inhibition; *Instruments:* NIRO-200, Hamamatsu Photonics K.K., Japan; *Cerebral area investigated*: left and right dorsolateral prefrontal (DLPFC); *Measure of Aerobic fitness*: maximal graded exercise test - VO_2_max was determined. | No intervention | - Strong activation of the DLPFC during RNG 1 s and 1.5 s compared to the count condition.  - DLPFC activation increased as a function of pace (difficulty) during the RNG task.  - Low fit older women showed significantly less DLPFC activation in the right hemisphere compared to their left hemisphere and compared to the high fit older women during the RNG task.  - Increases in [O_2_Hb] in the right DLPFC were found to mediate the relationship between VO_2_max level and executive performance during the RNG task at 1.5 s. |
| Cabral et al. (2017) | *Subjects*: male patient (mean age of 46) who has been alcohol and cigarette dependent for about 33 years; *Design study*: case report; *Task*: Stroop test; *Executive function*: interference control; *Instruments*: Imagent, ISS, Champaign, IL, USA; *Cerebral area investigated*: prefrontal cortex; *Measure of Aerobic fitness*: incremental protocol test - VO_2_max, ventilatory threshold and respiration compensation point were determinated. | Training program consisted of three weekly sessions during 90 days.  Gradual aerobic training: during first week, the patient was instructed to run 3and 6 min and the distance was recorded.  Running time was increased between 3 and 5 min every week to reach 40 to 50 min. | - At the end of training, the following parameters were improved: Time running (6 to 45 min); distance covered (765 to 8700 m); VO_2_max (24.2 to 30.1 mL^-1^.kg^-1^. min^-1^).  - Cognitive performance was improved (correct answers and reaction time) after 90 days.  - Prefrontal cortex oxygenation was increased after 90 days  - Cognitive performance improvement seemed to be matched with increased cerebral oxygenation. |
| Dupuy et al. (2015) | *Subjects*: 22 younger (24.6±3.6 years) and 36 older (62.9±5.4 years) women; *Design study*: cross sectional study; *Task*: trail making test, Modified Stroop task with two conditions (naming and execution condition); *Executive function*: cognitive flexibility and Interference control; Instruments: CW6, TechEn Inc., Milford, MA; *Cerebral area investigated*: prefrontal regions (left and right dorsolateral and left and right ventrolateral); *Measure of Aerobic fitness*: maximal continuous graded exercise test - VO_2_max was determined. | No intervention | - High fit women obtained better scores on the executive condition of the computerized Stroop task, than lower fit women.  - Only high fit women demonstrated a significant increase of [O_2_Hb] in the right inferior frontal gyrus (ventrolateral). |
| Goenarjo et al. (2020b) | *Subjects*: High-fit (22.8±4.2 years) and low-fit (24.8±5.1 years); *Design study*: cross sectional study; *Task*: dual-task (2-back + walking); *Executive function*: management of two or more tasks; *Instruments:* Portalite - Artinis Medical Systems, Elst, Netherlands; *Cerebral area investigated*: left and right dorsolateral and anterior prefrontal cortex; *Measure of Aerobic fitness*: maximal continuous graded exercise test - VO_2_peak was determined. | No intervention | - During 2-back, low-fit were significantly less accurate in dual-task condition, as compared to single-task condition, but there was no difference between conditions in high-fit.  -No significant correlation was found between VO_2_peak and dual-task cognitive performance.  -No significant correlation was found between VO_2_peak and ΔO_2_Hb.  -Significant correlation was found between VO_2_peak and ΔHHb on left and right prefrontal cortex. |
| Goenarjo et al. (2020a) | *Subjects*: active younger (21.8±2.0 years) and inactive younger (23.3±2.5 years); *Design study*: cross sectional study; *Task*: Stroop task (naming, inhibition and switching trials); *Executive function*: inhibition and cognitive flexibility; *Instruments:* Portalite - Artinis Medical Systems, Elst, Netherlands; *Cerebral area investigated*: left and right dorsolateral and anterior prefrontal cortex; *Measure of Aerobic fitness*: maximal continuous graded exercise test - VO_2_peak was determined. | No intervention | - Active younger were faster than inactive younger in switching condition but not in naming and inhibition conditions.  - Active younger had a greater ΔO_2_Hb than inactive younger in the switching condition.  - Between inhibition and switching condition, active younger showed greater ΔO_2_Hb in the right and left prefrontal cortex compared with inactive younger.  - Between naming and inhibition conditions, active younger showed greater ΔHHb in the right prefrontal cortex compared with inactive younger. |
| Kujach et al. (2018) | *Subjects*: 25 sedentary young (21.0±1.6 years); *Design study*: interventional study; *Task*: color-word matching Stroop; *Executive function*: interference control; *Instruments*: ETG-7000, Hitachi Medical Corporation, Japan; *Cerebral area investigated*: left and right dorsolateral, ventrolateral and frontopolar cortex; *Measure of Aerobic fitness*: maximal oxygen consumption test - maximal aerobic power was determinated. | Modified Gibala’s group’s High-Intensity Intermittent Exercise: Eight repetitions of 30 s of ergometer exercise at 60% of maximal aerobic power at 100 rpm and 30 s resting after two minutes of warm-up (50 Watts) at 60 rpm. | - Acute high-intensity intermittent exercise improved interference control.  - Hemodynamic activity of left-dorsolateral & prefrontal cortex correlated with interference control during acute high-intensity intermittent exercise in subjects with good cardiorespiratory fitness (40.5 mL^-1^.kg^-1^. min^-1^). |
| Ludyga et al. (2019) | *Subjects:* 20 High-fit (17.2±1.0 years) and 20 Low-fit (17.0±1.1 years); *Design study*: cross sectional study; *Task*: modified Stroop color-word test; *Executive function*: Interference control (inhibition); *Instruments*: NIRSport, NIRx Medical Technologies, Berlin, Germany; *Cerebral area investigated*: left and right dorsolateral prefrontal cortex; *Measure of Aerobic fitness*: PWC170 cycle test - relative power output was determinated. | No intervention | - Lower interference control in high-fit compared low-fit.  - Inverse relation between aerobic fitness and behavioural performance.  - Hemodynamic activity of prefrontal cortex did not explain the relation of aerobic fitness with behavioural performance. |
| Mekari et al. (2019) | *Subjects*: 66 (44 females) older adults (68±6.3 years); *Design study*: cross sectional study; *Task*: Trail Making Test; *Executive function*: cognitive flexibility; *Instruments*: Portalite, Artinis Medical Systems, Netherlands; *Cerebral area investigated*: Left prefrontal cortex; *Measure of Aerobic fitness*: maximal continuous graded exercise test - peak power output was used as a marker of cardiorespiratory fitness. | No intervention | - Increased cardiorespiratory fitness was associated with better performance during Trail Making Test B.  - Cerebral oxygenation in higher fit older adults mediated the relationship with improved executive functioning.  - Particularly, in older adults with higher cardiorespiratory fitness, cerebral oxygenation was related to executive functioning. |

ΔHHb: Changes in deoxyhemoglobin concentration; ΔO_2_Hb: Changes in oxyhemoglobin concentration; [HHb]: oxyhemoglobin concentration; [O_2_Hb]: deoxyhemoglobin concentration
